# *PAX3* haploinsufficiency in Maine Coon cats with dominant blue eyes and hearing loss resembling the human Waardenburg syndrome

**DOI:** 10.1093/g3journal/jkae131

**Published:** 2024-06-13

**Authors:** Gabriela Rudd Garces, Daniela Farke, Martin J Schmidt, Anna Letko, Katja Schirl, Marie Abitbol, Tosso Leeb, Leslie A Lyons, Gesine Lühken

**Affiliations:** Institute of Animal Breeding and Genetics, Justus Liebig University Giessen, 35390 Giessen, Germany; Generatio GmbH, 69115 Heidelberg, Germany; Clinic for Small Animals, Neurosurgery, Neuroradiology and Clinical Neurology, Justus Liebig University Giessen, 35392 Giessen, Germany; Clinic for Small Animals, Neurosurgery, Neuroradiology and Clinical Neurology, Justus Liebig University Giessen, 35392 Giessen, Germany; Vetsuisse Faculty, Institute of Genetics, University of Bern, 3012 Bern, Switzerland; Department of Molecular Biology, LABOKLIN GmbH & Co. KG, 97688 Bad Kissingen, Germany; Université Claude Bernard Lyon, VetAgro Sup, 69280 Marcy-l’Etoile, France; Institut NeuroMyoGène INMG-PNMG, CNRS UMR5261, INSERM U1315, Faculté de Médecine, Université Claude Bernard Lyon 1, Rockefeller, 69008 Lyon, France; Vetsuisse Faculty, Institute of Genetics, University of Bern, 3012 Bern, Switzerland; Department of Veterinary Medicine and Surgery, College of Veterinary Medicine, University of Missouri, Columbia, MO 65211, USA; Institute of Animal Breeding and Genetics, Justus Liebig University Giessen, 35390 Giessen, Germany

**Keywords:** *Felis catus*, whole-genome sequence, neural development, melanocyte, deafness, pigmentation, precision medicine, animal model

## Abstract

This study investigated the dominant blue eyes (DBE) trait linked to hearing impairment and variable white spotting in Maine Coon cats. Fifty-eight animals descending from 2 different DBE lineages, the Dutch and the Topaz lines, were sampled. They comprised 48 cats from the Dutch bloodline, including 9 green-eyed and 31 blue-eyed cats, with some individuals exhibiting signs of deafness, and 8 stillborn kittens. Samples from the Topaz lineage included 10 blue-eyed animals. A brainstem auditory evoked response test revealed a reduced to absent response to auditory stimuli and absent physiological waveforms in all of the 8 examined DBE animals. We sequenced the genome of 2 affected cats from the Dutch line and searched for variants in 19 candidate genes for the human Waardenburg syndrome and pigmentary disorders. This search yielded 9 private protein-changing candidate variants in the genes *PAX3*, *EDN3*, *KIT*, *OCA2*, *SLC24A5*, *HERC2*, and *TYRP1*. The genotype–phenotype cosegregation was observed for the *PAX3* variant within all animals from the Dutch lineage. The mutant allele was absent from 461 control genomes and 241 additionally genotyped green-eyed Maine Coons. We considered the *PAX3* variant as the most plausible candidate—a heterozygous nonsense single base pair substitution in exon 6 of *PAX3* (NC_051841.1:g.205,787,310G>A, XM_019838731.3:c.937C>T, XP_019694290.1:p.Gln313*), predicted to result in a premature stop codon. *PAX3* variants cause auditory–pigmentary syndrome in humans, horses, and mice. Together with the comparative data from other species, our findings strongly suggest *PAX3*:c.937C>T (OMIA:001688-9685) as the most likely candidate variant for the DBE, deafness, and minimal white spotting in the Maine Coon Dutch line. Finally, we propose the designation of *DBE^RE^* (Rociri Elvis Dominant Blue Eyes) allele in the domestic cat.

## Teaser

In this study, Rudd Garces *et al*. investigated two lineages of DBE Maine Coon cats, the Dutch and the Topaz lines. Some of these cats are deaf. After whole-genome sequencing of two DBE cats, the authors identified a nonsense variant in PAX3 (g.205,787,310G>A). The mutant allele was absent from 461 feline genomes, and genotypes at the PAX3:c.937C>T variant perfectly co-segregated with DBE, deafness and minimal white spotting in the Dutch line cats, however it was not found in DBE cats of the Topaz lineage.

## Introduction

Waardenburg syndrome (WS; Waardenburg 1951) is a genetic auditory–pigmentary disorder in humans characterized by anomalies in hair, eye, and skin pigmentation, as well as sensorineural hearing impairment. Hair pigmentary anomalies encompass a white forelock and premature graying, while iris changes manifest as heterochromia iridis and/or striking blue eyes. Skin pigmentation anomalies predominantly consist of depigmented patches (Read and Newton 1997; Pingault *et al.* 2010). The interplay of hearing loss and pigmentary abnormalities of WS results from an abnormal proliferation, survival, migration, or differentiation of melanoblasts and/or melanocytes derived from the neural crest during embryonic development (Pingault *et al.* 2010). Additional clinical features may include upper limb skeletal deformities; neurological abnormalities such as mental impairment, myelination defects, and ataxia; and Hirschsprung disease (Pingault *et al.* 1998; Tekin *et al.* 2001; Bondurand *et al.* 2007; Tamayo *et al.* 2008). Due to its clinical and genetic heterogeneity, WS is classified into 4 primary phenotypes comprising diverse subtypes. The Online Mendelian Inheritance in Man (OMIM) database (https://omim.org/) currently lists 10 WS types (OMIM PS193500) with 8 having known pathogenic variants in 6 genes (Table 1).

**Table 1.** Functional candidate genes for WS in humans.

Gene	Full name	WS type	Mode of inheritance	OMIM identifier
*PAX3*	Paired box 3	WS1, WS3	AD or AR	193500, 148820
*MITF*	Melanocyte inducing transcription factor	WS2A	AD	193510
*SOX10*	SRY-box transcription factor 10	WS2E, WS4C	AD	611584, 613266
*KITLG*	KIT ligand	WS2F	AR	619947
*EDN3*	Endothelin 3	WS4B	AD or AR	613265
*EDNRB*	Endothelin receptor type B	WS4A	AD or AR	277580

AD, autosomal dominant; AR, autosomal recessive.

The molecular investigation of spontaneous pigmentation disorders and concurrent deafness has been a prominent area of study in domestic animals. Similar to WS in humans, the phenotypes observed in animals show locus heterogeneity and different modes of inheritance. In bovines, coding variants within the microphthalmia-associated transcription factor (*MITF*) gene cause distinct white coat color phenotypes (Petersen *et al.* 2023; OMIA 001401-9913), often associated with ocular malformations such as microphthalmia (Wiedemar and Drögemüller 2014; OMIA 001931-9913), bilateral hearing loss, heterochromia iridis, and glass-eyed albino phenotype (Philipp *et al.* 2011; Bourneuf *et al.* 2017; OMIA 001680-9913). The Asian swamp buffalo exhibits a white-spotted coat color and blue eyes, a result of two dominant mutant alleles within the *MITF* gene, including a nonsense variant and a donor splice site variant (Yusnizar *et al.* 2015; OMIA 000214-89462). Recessive mutant alleles in the *MITF* gene are also responsible for bilateral deafness, blue/pale eyes, and absent skin pigmentation in Rongchang pigs and American mink (Chen *et al.* 2016; Manakhov *et al.* 2019; OMIA 001401-9823; OMIA 001680-452646). In horses, over 10 independent variants in the *MITF* (OMIA 000214-9796) and *PAX3* (OMIA 001688-9796) genes explain the splashed white phenotype, frequently accompanied by blue eyes and, in some cases, deafness (Hauswirth *et al.* 2012, 2013; Dürig *et al.* 2017; Henkel *et al.* 2019; Magdesian *et al.* 2020; Patterson Rosa *et al.* 2022; Bellone *et al.* 2023; McFadden *et al.* 2023). Additionally, the equine overo coat color pattern, characterized by pigment spreading down both sides from the dorsal midline and, in some instances, blue eyes, is caused by a heterozygous semidominant variant in the endothelin receptor type B (*EDNRB*) gene. In its homozygous state, this allele leads to the overo lethal white foal syndrome (OLWFS), characterized by aganglionosis, a white or nearly white coat, blue irises, and a high incidence of deafness (Metallinos *et al.* 1998; Santschi *et al.* 1998; Yang *et al.* 1998; Magdesian *et al.* 2009; OMIA-000629-9796). An additional large structural variant, resulting in the complete loss of the *EDNRB* gene, is the cause of a lethal recessive hypopigmentation syndrome in Cameroon sheep (Lühken *et al.* 2012; Pauciullo *et al.* 2013; OMIA 001765-9940). Similar to OLWFS, homozygous lambs are white and blue-eyed.

In domestic cats, sensorineural deafness and the presence of blue eyes have been associated with dominant white coat color in both purebred and mixed-breed animals (Strain 2007; Cvejic *et al.* 2009; Annemarie *et al*. 2022). The dominant white locus (*W*) exhibits pleiotropic effects, showing complete penetrance for absence of coat pigmentation and incomplete penetrance for deafness and iris hypopigmentation (Kaelin and Barsh 2013). The genetic basis of this phenotype involves a 623-bp insertion of a long terminal repeat (LTR) fragment of a feline endogenous retrovirus (FERV1) into intron 1 of the *KIT* gene for dominant white (*W allele*) and a full length 7,125-bp FERV1 insertion for white spotting (*w^s^ allele*) at the same position (David *et al.* 2014; Frischknecht *et al.* 2015; OMIA 000209-9685; OMIA:001737-9685).

Since the mid-1990s, selective breeding for blue eyes and minimal white spotting has led to the development of the Altai, Topaz, and Celestial cat breeds in Europe. Pedigree data confirmed autosomal dominant inheritance pattern for the dominant blue eyes (DBE). This trait has also been incorporated into various breeds that traditionally had common yellow, copper, or green eye colors such as British Shorthair and Longhair, Siberian, Persian, Sphynx, and Maine Coon. Notably, some cats from DBE lines assumed to carry the causative allele do not exhibit blue eyes; these animals are referred by the breeders as latent (http://messybeast.com/blue-eye-breeds.htm, accessed on 8 May 2024).

In Maine Coon cats, breeders have identified 4 primary DBE lines: the Dutch line (Rociri Elvis founder), the Topaz line (Roxi and Seymour founders, mix of 2 DBE lines, one of which is the Altai line), the Pillowtalk line (common ancestor with Rociri Elvis), and the Nahal line originating from a DBE domestic cat in Russia. The Dutch and Topaz alleles have contributed to the establishment of multiple DBE catteries in Europe and North America (http://messybeast.com/DBE-maine-coon.htm, accessed on 8 May 2024). However, the molecular genetic basis underlying the feline DBE with minimal white spotting remained unknown. Therefore, the present study aimed to characterize a new form of hereditary auditory–pigmentary disorder in Maine Coon cats and elucidate the underlying genetic etiology.

## Materials and methods

### Ethics approval statement

All cats in this study were privately owned and were examined for diagnostic purposes with the consent of the owner and handled according to good ethical standards. Collection of animal samples was approved by the Veterinary Department of the Regional Council of Giessen (19 c 20 15 h 02 Gi 19/1 KTV 22/2020).

### Animals

This study included 58 Maine Coons from 2 blue-eyed lineages: Dutch and Topaz lines. We obtained EDTA blood and buccal swab samples from 48 cats from the Dutch line, including 9 green-eyed and 31 blue-eyed cats and 8 stillborn. They originated from Germany (*n* = 36), Italy (*n* = 8), and the United Kingdom (*n* = 4). Samples from the Topaz line included 10 DBE individuals, 9 cats of Italian origin and 1 animal from Russia (Supplementary Table 1). We additionally used DNA samples from 241 unrelated green-eyed Maine Coons originating from various regions in Europe and the United States available from the biobank of the Institute of Genetics, University of Bern.

### Clinical examination and brainstem auditory evoked response testing

Thirteen related cats from the Dutch lineage originating from the same German cattery were brought to the Giessen University clinic for a general physical examination. Brainstem auditory evoked responses (BAER) were performed under sedation in 10 animals. BAER testing was performed in both ears by using an electrodiagnostic unit (Nicolet Fusion). Subcutaneous stainless steel electrodes were placed as follows: the positive electrode at the vertex, the negative electrode at the level of the stylomastoid process, and the ground electrode at the level of the neck. The auditory stimulus was given as clicks with a duration of 0.2 ms delivered via ear probes at a rate of 30/s and intensity of 75, 90, and 105 dB normal hearing level. A masking noise to the contralateral ear was delivered. The BAER was obtained by averaging 300 recordings of 10 ms. Filters were set at the cutoff frequencies of 100 Hz and 3 kHz. A normal hearing ability was diagnosed if waves I–V were visible at 75, 90, and 105 dB in the traces from both ears, unilaterally deaf if an absence of physiological waveforms (flatline) was observed in 1 ear, and bilaterally deaf if a flatline was obtained from both ears.

### DNA extraction

Genomic DNA was isolated from EDTA blood and buccal swab samples (sterile transport swabs; COPAN Italia SpA, Brescia, Italy; GenoTube, Life Technologies, Darmstadt, Germany) using the NucleoSpin Blood Kit (Macherey-Nagel, Düren, Germany) and the Gentra Puregene Tissue Kit (QIAGEN GmbH, Hilden, Germany), respectively.

### Whole-genome sequencing and variant analyses

Illumina TruSeq DNA PCR-Free libraries were prepared for 2 blue-eyed, deaf, and white-spotted cats from the Dutch line of the German cattery. We collected 306 million (WGS 1) and 323 million (WGS 2) 2× 150-bp paired-end reads on a NovaSeq 6000 instrument (average of 20× read depth). First, the reads were quality controlled with fastp v0.23.2 using the flags –cut_window_size 4 –qualified_quality_phred 20 –length_required 50 (Chen *et al.* 2018). The reads were mapped using BWA v0.7.17 (Li and Durbin 2009) and sorted using Samtools v1.10 (Li *et al.* 2009) to the latest feline reference genome assembly F.catus_Fca126_mat1.0. Before performing variant calling, duplicated variants were marked using the function MarkDuplicates of the Picard Tools v2.21.8 (https://broadinstitute.github.io/picard/). Variant calling of single-nucleotide variants (SNVs) and small indels was performed using HaplotypeCaller from GATK v4.1.3.0 (DePristo *et al.* 2011). The two individual GVCF files were combined and genotyped using the tools CombineGVCFs and GenotypeGVCFs from GATK v 4.1.3.0 (DePristo *et al.* 2011), respectively, in order to receive 1 comprehensive VCF file. Finally, functional effect prediction for all the identified variants was performed with SnpEff v4.3 (Cingolani *et al.* 2012) using NCBI annotation release 105 (https://www.ncbi.nlm.nih.gov/genome/annotation_euk/Felis_catus/105/). The whole-genome sequencing (WGS) data were submitted to the European Nucleotide Archive with the study accession PRJEB64577 and sample accessions SAMEA114193917 (WGS 1) and SAMEA114193918 (WGS 2; Supplementary Table 2).

For variant filtering, a hard filtering approach was employed, which required to identify variants in which the 2 affected cats were either homozygous for the alternative allele (1/1) or heterozygous (0/1) across 19 candidate genes, 6 associated with human WS and 13 with pigmentary anomalies—without involving anomalies in other organ systems rather than the auditory (Table 1; Supplementary Table 3). Protein-changing variants with high and moderate impact according to SnpEff v4.3 (Cingolani *et al.* 2012) were prioritized. The output of the variant filtering is shown in Supplementary Table 4.

In addition, we extracted the genotypes at the identified protein-changing variants from 2 publicly available data sets as described previously (Rudd Garces et al 2021). One data set comprised 57 cat genomes with European origin from the Institute of Genetics, University of Bern (Supplementary Table 2), and the other contained 404 cat genomes, with a significant portion of samples originating from North America, from the 99 Lives Consortium (Buckley *et al.* 2020). These data sets included 58 Maine Coons and 403 purebred and mixed-breed individuals. Sequences for the cat analysis of the 99 Lives project are available under BioProject accession IDs PRJNA308208 and PRJNA288177 at the sequence read archive (https://www.ncbi.nlm.nih.gov/sra).

Since our variant calling pipeline considered only small variants comprising SNVs and indels of up to ∼25 nucleotides, we performed an additional visual inspection to exclude any large structural variants in coding, noncoding, upstream, and downstream regions (up to 1,000 bp) within the 19 candidate genes using the Integrative Genomics Viewer (IGV; Robinson *et al.* 2011).

### Genotyping of the candidate variants

Numbering within the feline *PAX3* gene corresponds to the NCBI RefSeq accession numbers XM_019838731.3 (mRNA) and XP_019694290.1 (protein).

We used Sanger sequencing to genotype the variant *PAX3*:XM_019838731.3:c.937C>T. PCR products were amplified from genomic DNA using GoTaq G2 Flexi DNA Polymerase (Promega, Madison, WI, USA) together with forward and reverse primers. PCR amplicons were purified using a commercial kit (MSB Spin PCRapace, Stratec Molecular, Berlin, Germany) and sent to LGC Genomics GmbH (Berlin, Germany) or the Institute of Genetics, University of Bern, for Sanger sequencing. Sequences were analyzed using the Chromas 1.74 software (Technelysium Pty Ltd, South Brisbane, Australia). The primer sequences used for this experiment are given in Supplementary Table 5.

### Genotyping of dominant white alleles

Genotypes at the *KIT* variants for dominant white (OMIA:000209-9685) and white spotting (OMIA:001737-9685) were commissioned from LABOKLIN GmbH & Co. KG (Bad Kissingen, Germany).

## Results

### Clinical investigations and phenotype description

A Maine Coon breeder reported multiple deaths in several litters from 2020 to 2023 and observed signs of deafness in juvenile and adult cats with blue eyes. Upon pedigree analysis, a common ancestral lineage for DBE was identified, the Dutch line. This prompted the beginning of a genetic investigation.

**Fig. 1.**
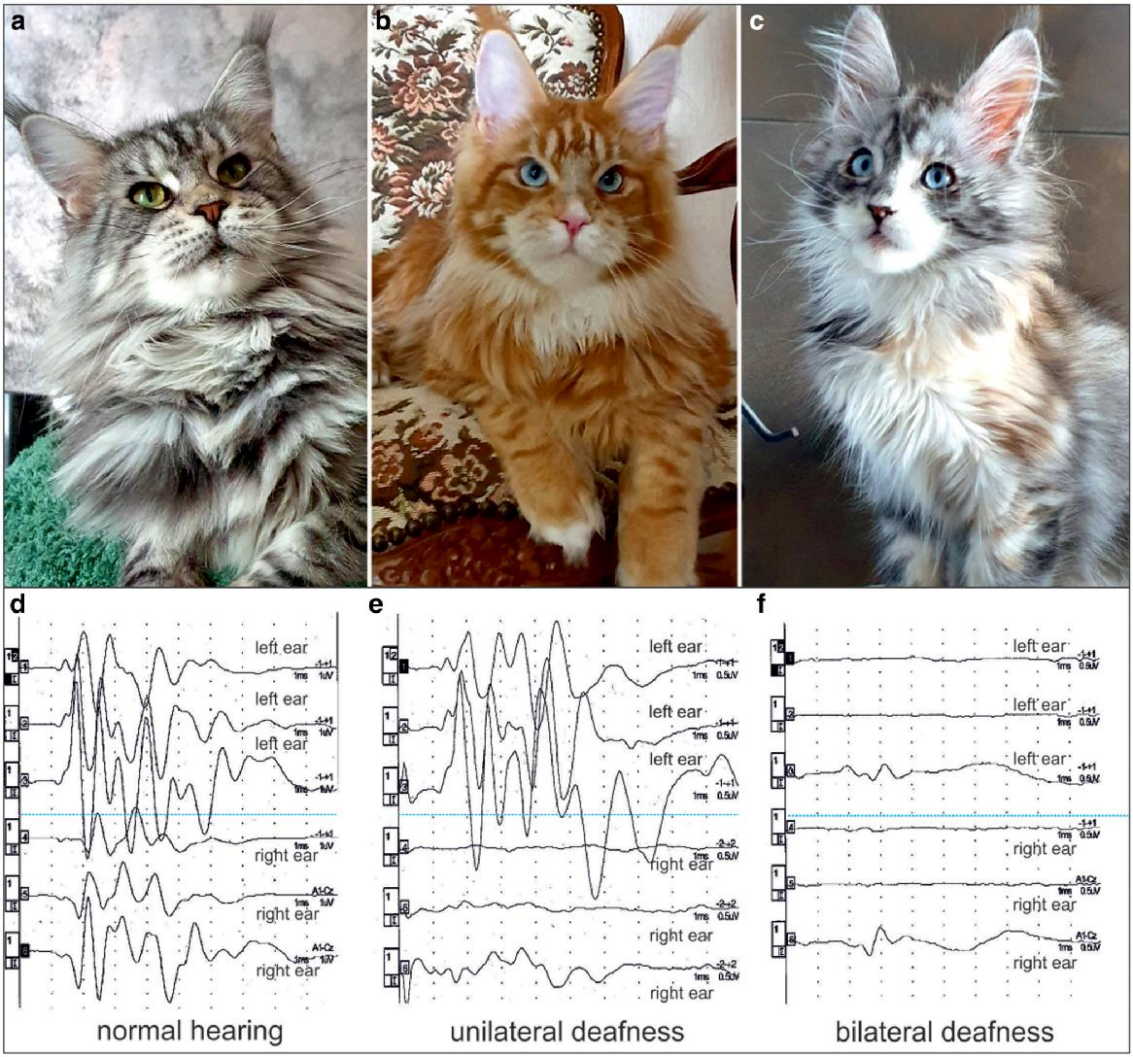
Phenotypes and auditory function in the investigated Maine Coon cats. a) Individual exhibiting normal iris pigmentation (green eyes) and fully pigmented skin. b and c) WGS animals showing dominant blue eyes and minimal white spotting. The white spot on the bridge of the nose together with pink nasal planum and pink lips b) and the white stripe on the left side of the nose c) indicate a lack of facial skin melanocytes. To assess auditory function, BAER d–f) were recorded. The BAER comprises five distinctive waves corresponding to the transmission of auditory stimuli along the central hearing pathway. Repetitive auditory stimulation of both ears yields symmetrical waves in cats with normal hearing abilities a and d). A complete flat line or arbitrary “noise waves” indicate the absence of electrical activity transmission from the inner ear (cochlea) to the rest of the auditory pathway, leading to a diagnosis of sensorineural deafness. This condition may manifest unilaterally (right sensorineural deafness b and d) or bilaterally (bilateral sensorineural deafness c and f).

From this breeder, we obtained samples of 36 related animals—according to their genealogical origin—primarily from Germany. Eight cats had green eyes, while 20 were blue-eyed (Supplementary Table 1). There were 8 stillborn kittens from 3 litters that, according to the breeder, exhibited yellow spots, distended bellies, cramps, dehydration, and abnormal skulls. There was no available information regarding the eye and coat colors of the stillborn kittens. Additionally, 2 juvenile kittens died a few months after birth. One had a cleft palate and was euthanized, while the other died, apparently due to an infection. Furthermore, 13 cats of this German cattery underwent a general clinical examination, which was normal in all cats. BAER testing was conducted in 10 animals, 2 green-eyed and 8 blue-eyed. Green-eyed cats had physiological waveforms in BAER testing (Fig. 1a and d). Abnormal waveforms were found in all blue-eyed cats, among which 3 showed unilateral sensorineural deafness (Fig. 1b and e), while 5 exhibited bilateral sensorineural deafness (Fig. 1c and f).

We extended our investigation to further DBE catteries, obtaining 11 additional samples from Dutch line cats including 10 blue-eyed and 1 green-eyed animal. Furthermore, samples from the Topaz lineage included 9 related animals and 1 unrelated cat—according to their pedigree records—all blue-eyed. None of these cats were available for clinical examination.

The coat color pattern exhibited variability within the Dutch and Topaz lineages. Most cats had fur colors ranging from black, red, blue, cream with or tabby marks, with or without silver/smoke modification, with varying degrees of white spotting—from large patches on the chest, head, and paws to white stripes. Conversely, 8 cats exhibited solid and tabby fur coloration and patterns without white spotting. Most of the blue-eyed cats presented varying degrees of white spotting except for 2 individuals (see Supplementary Fig. 2), while the majority of green-eyed animals showed fully pigmented skin except for 2 littermates from the Dutch line (see Supplementary Fig. 1). There was no dominant white cat among the investigated cohort. Detailed phenotype information on the 58 sampled cats in this study is provided in Supplementary Table 1.

### Genetic analyses

Given the observed segregation of the phenotype and the analysis of the available pedigree in the studied cat cohort, an autosomal dominant mode of inheritance, exhibiting pleiotropic effects, with complete penetrance for blue eyes and incomplete penetrance for deafness and white spotting was assumed. Subsequently, driven by the hypothesis of a breed-specific rare deleterious variant responsible for DBE, deafness, and minimal white spotting resembling the human WS, the whole genomes of 2 blue-eyed cats, a male exhibiting unilateral deafness (Fig. 1b; WGS 1) and a female exhibiting bilateral deafness (Fig. 1c; WGS 2), were sequenced. The variant calling pipeline detected more than 5 million homozygous and more than 7 million heterozygous variants in each animal. To refine our search, we focused on variants in the 6 known WS candidate genes and 13 other genes for pigmentary anomalies (Supplementary Table 4). As a result, we pinpointed 9 private protein-changing candidate variants in the genes *PAX3*, *EDN3*, *KIT*, *OCA2*, *SLC24A5*, *HERC2*, and *TYRP1* (Table 2).

**Table 2.** Details of 9 protein-changing variants in 7 candidate genes detected in the whole genomes of 2 affected cats.

Chr.	Position	Ref.	Alt.	Gene	HGVS-c	HGVS-p	Allele frequency in 461 control genomes
A3	4,046,887	G	C	*EDN3*	c.400C>G (XM_023251053.2)	p.Pro134Ala	0.96
B1	161,352,103	T	TC	*KIT*	c.95-1_95insG (NM_001009837.3)		1
C1	205,787,310	G	A	*PAX3*	c.937C>T (XM_019838731.3)	p.Gln313*	0
B3	55,658,393	T	A	*SLC24A5*	c.859A>T (XM_011282858.4)	p.Ser287Cys	0.06
B3	27,704,283	A	G	*OCA2*	c.235T>C (XM_003986906.5)	p.Phe79Leu	0.97
B3	27,869,698	T	C	*HERC2*	c.5512A>G (XM_045058958.1)	p.Ile1838Val	0.97
B3	27,900,565	T	C	*HERC2*	c.3073A>G (XM_045058958.1)	p.Ile1025Val	0.97
D4	38,129,873	G	C	*TYRP1*	c.8G>C (NM_001042560.2)	p.Gly3Ala	0.85
D4	38,129,957	C	T	*TYRP1*	c.92C>T (NM_001042560.2)	p.Ala31Val	0.45

Genotypes at these 9 variants were extracted from 461 cat genomes of 2 publicly available data sets. Significant prevalence was observed for variants within the *EDN3*, *OCA2*, *HERC2*, and *TYRP1* genes (Table 2). Conversely, the *KIT* variant most likely represents a technical artifact due to an error in the reference genome assembly. Finally, the *SLC24A5* allele was found in cats that did not have DBE phenotype. Hence, these variants were ruled out as candidate causatives.

**Fig. 2.**
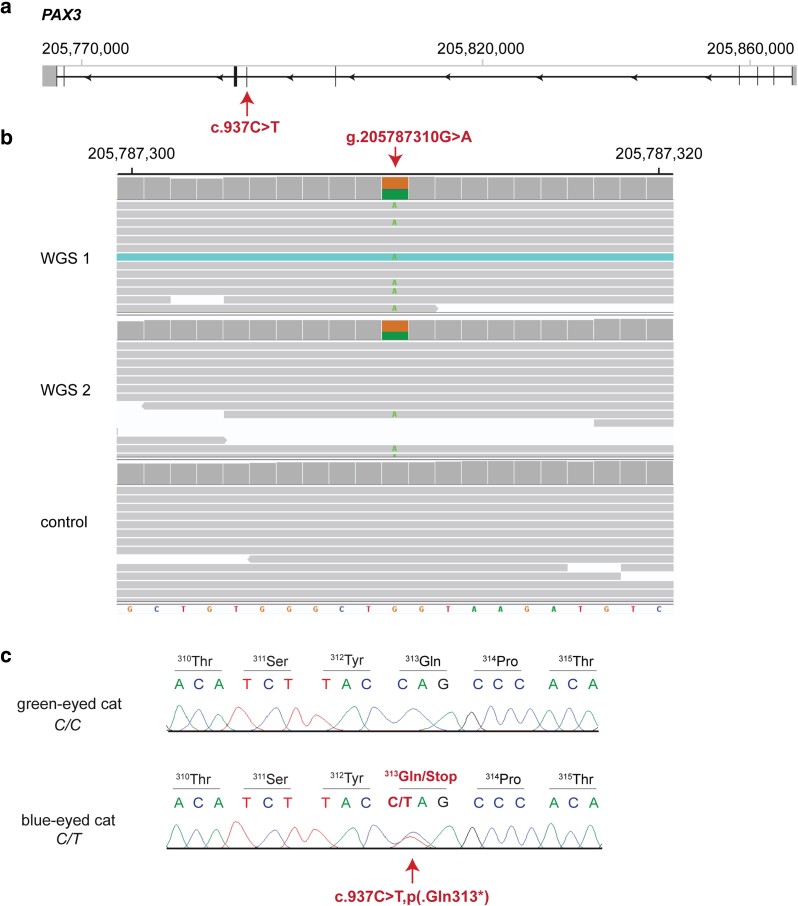
Details of the *PAX3*:c.937C>t variant in Maine Coon cats with blue eyes and hearing loss. a) Schematic representation of the *PAX3* gene showing the WGS-detected variant location in exon 6 (the scheme corresponds to transcript XM_019838731.3). b) IGV screenshot showing the short-read alignments (average of 20× read depth) of 2 DBE cats and an unrelated control Maine Coon cat at the position of the variant. c) Representative Sanger electropherograms of blue-eyed and green-eyed cats are shown. Amino acid translations are indicated. The premature stop codon of the mutant sequence is indicated in red.

In contrast, none of the cat control genomes presented the mutant *PAX3* allele; consequently, we prompted this variant as the most plausible candidate. It represents a heterozygous nonsense variant in the sixth exon of *PAX3*, XM_019838731.3:c.937C>T, and is predicted to result in a premature stop codon, XP_019694290.1:p.(Gln313*). Consequently, 35% of the 484 codons of the wild-type Paired box 3 transcription factor would be truncated. The genomic designation of this variant is ChrC1:205,787,310G>A (F.catus_Fca126_mat1.0; Fig. 2).

We genotyped the *PAX3*:c.937C>T variant in the investigated Maine Coon cohort. All 31 blue-eyed cats of the Dutch lineage were heterozygous for the mutant allele, while all 9 green-eyed cats were homozygous wild type. Among the 8 stillborn kittens without eye color information, 6 were heterozygous and 2 were homozygous wild type. The segregation of the genotypes was compatible with a monogenic autosomal dominant mode of inheritance for DBE, deafness, and minimal white spotting in the Dutch line. Within the Topaz line, the mutant *PAX3* allele was absent in all the studied animals. Furthermore, the variant was genotyped in 241 additional unrelated green-eyed Maine Coon cats, and the mutant allele was absent in all the tested control animals (Table 3).

**Table 3.** Genotype association of the *PAX3* and *KIT* variants with DBE, deafness, and white spotting in 299 cats (for coat color patterns of the studied cats, see Supplementary Table 1).

Phenotype	*PAX3* genotype	*KIT* genotype (*W*, *w^s^*, *w*)
Dutch line DBE cats (no BAER tested)	*wt*/*mut n* = 23	*w*/*w n* = 11 *w^s^*/*w n* = 9 *w^s^*/*w^s^ n* = 2 Not genotyped *n* = 1
Dutch line DBE deaf cats (BAER tested)	*wt*/*mut n* = 8	*w*/*w n* = 5 *w^s^*/*w n* = 3
Dutch line green-eyed cats	*wt*/*wt n* = 9	*w*/*w n* = 7 *w^s^*/*w n* = 2
Dutch line stillborn kitten	*wt*/*wt n* = 2 *wt*/*mut n* = 6	*w*/*w n* = 2 *w^s^*/*w n* = 6
Topaz line DBE cats (no BAER tested)	*wt*/*wt n* = 10	*w*/*w n* = 4 *w^s^*/*w n* = 3 Not genotyped *n* = 3
Control Maine Coon cats (green-eyed)	*wt*/*wt n* = 241	Not genotyped

Given that several of the investigated DBE Maine Coons presented various degrees of white spotting, a test for the known dominant white (*W*) and white spotting (*w^s^*) alleles was conducted. While no cat carried the dominant *W* allele, 23 animals were heterozygous for the *w^s^* allele, 2 were homozygous mutant, and 29 were wild type. Four samples were not genotyped. The *w^s^* allele was observed in both blue-eyed and green-eyed cats. Notably, 20 DBE cats, 5 of them with compromised hearing, did not have 2 of the known *KIT* variants associated with white patterns and blue eyes in cats. However, all of them presented white spotting. This evidences that the presence of a single copy of the mutant *PAX3* allele is sufficient to cause DBE, sensorineural hearing loss, and minimal white spotting (Table 3; Supplementary Table 1).

Finally, no discernible structural alterations in the genomic sequences were identified.

## Discussion

In this study, we present a detailed clinical and genetic analysis of an inherited dominant phenotype characterized by iris hypopigmentation and incomplete penetrance deafness and minimal white spotting in Maine Coon cats. We extended our investigation to explore the presence of the identified underlying genetic defect in 2 blue-eyed lineages of Maine Coon, the Dutch and Topaz lines.

The investigated phenotype partially resembles the human WS1, which is characterized by pigmentary abnormalities of the hair and iris, sensorineural hearing loss, and dystopia canthorum. Unlike humans with WS1, dystopia canthorum was not observed in the studied Maine Coon cats, and facial white spotting was noted in both green-eyed (Supplementary Fig. 1) and blue-eyed cats. Even though 16 DBE cats of the Dutch line and 4 cats of the Topaz line were negative for the *KIT* variants that influence the *Spotting* and *White* loci phenotypes. Like human patients, blue-eyed cats showed bilateral or unilateral sensorineural deafness, while green-eyed animals exhibited normal hearing ability. Deafness has been largely documented in blue-eyed white cats and is caused due to a lack of melanocytes within the stria vascularis of the inner ear, responsible for maintaining a high potassium concentration within the endolymph. This is crucial for generating endocochlear potentials within the hair cells and translating sound waves into electrical potentials (Takeuchi *et al.* 2000).

Using a functional candidate gene approach, along with WGS, we identified the *PAX3*:c.937C>T nonsense variant as the most likely causative variant for the investigated phenotype in cats of the Dutch line. Thus, we propose that this variant represents the *DBE^RE^* (Rociri Elvis dominant blue eye) allele. The cause of death in the 8 stillborn kittens could not be determined but was not correlated with their *PAX3* genotype, and the cats were not homozygous for the *PAX3* variant.

Paired box 3 is a transcription factor of the PAX family characterized by a highly conserved DNA binding domain (paired box). It is expressed during development and plays critical roles in the proper formation of the central and peripheral nervous systems, the morphogenesis of the outflow tract region of the heart, and the muscular system (Epstein 2000). In later developmental stages, PAX3 is expressed by various cell types and structures originating from the neural crest, including melanoblasts. PAX3 has a crucial role in the differentiation of melanoblasts into melanocytes by regulating, together with *SOX10*, the expression of *MITF* (Bondurand *et al.* 2000; Lang *et al.* 2005). Additionally, PAX3-expressing neural crest–derived cells contribute to the formation of diverse structures, such as the inner ear, mandible, and maxilla (Epstein 2000). *PAX3* involvement in controlling a wide array of developmental events is facilitated by alternative splicing, resulting in transcripts encoding isoforms with different C-termini (Barber *et al.* 1999). *PAX3* heterozygous loss-of-function variants have been identified as causative for auditory–pigmentary disorders in humans, horses, and mice models, but have so far not been reported in domestic cats.

The splotch (*Sp*) mouse mutant is a model for Pax3 loss-of-function studies and WS1. Effects on homozygotes for *Pax3* variants vary in severity, including embryonic to perinatal death and malformations of neural tube, spinal ganglia, heart, vertebral column, hindbrain, and limb musculature. In contrast, heterozygous *Sp^+/−^* mice exhibit white belly spots and variable spotting on the back and extremities (Epstein *et al.* 1991; Li *et al.* 1999). Unlike WS1 human patients, *Sp^+/−^* mice do not show alterations in auditory function and ear morphology when compared with wild-type animals (Steel and Smith 1992; Buckiová and Syka 2004).

The human ClinVar database (Landrum *et al.* 2014) lists over 30 pathogenic variants in *PAX3* causing WS1 and WS3 (OMIM 193500 and OMIM 148820, respectively). While both WS forms share most clinical features, WS3 patients often additionally exhibit upper limb abnormalities. Notably, although most patients have only 1 mutant *PAX3* allele, 2 individuals with biallelic loss-of-function variants were identified, surviving at least into early infancy without neural tube defects (Zlotogora *et al.* 1995; Wollnik *et al.* 2003). This finding is intriguing, considering that in mutant mice, homozygosity typically leads to severe neural tube defects and intrauterine or neonatal death. Other *PAX3* variants cause craniofacial–deafness–hand syndrome (CDHS), which is occasionally classified as a subtype of WS (OMIM 122880). CDHS is an autosomal dominant disorder characterized by dysmorphic facial features, hand abnormalities, absent or hypoplastic nasal and wrist bones, and severe sensorineural hearing impairment (Asher *et al.* 1996; Sommer and Bartholomew 2003). Additionally, the human alveolar rhabdomyosarcoma can result from fusion of *PAX3* with the *FOXO1* gene due to a chromosomal translocation (RMS2; OMIM 268220; Anderson *et al.* 2001).

In horses, the splashed white phenotype, accompanied by blue eyes or iris heterochromia, is attributed to 3 dominant deleterious alleles (*SW2*, *SW4*, and *SW10*) in the *PAX3* gene. Some of the horses were reported to be deaf; however, the hearing status of the *PAX3* mutant animals was not consistently evaluated (Hauswirth *et al.* 2012, 2013; McFadden *et al.* 2023; OMIA 001688-9796).

The *PAX3* nonsense variant, c.937C>T, p.Gln313*, in the Maine Coon cats of this study leads to a premature stop codon. Drawing from the existing knowledge on *PAX3* heterozygous variants and their functional impact in humans, mice, and horses, we consider that the resulting haploinsufficiency in *PAX3* leads to the observed phenotype in the Maine Coon cats. Although we did not establish functional proof for the causality of the *PAX3* variant, we have gathered sufficient ancillary evidence to assert its causality. Applying the ACMG/AMP consensus criteria for human diagnostics (Richards *et al.* 2015) to the feline *PAX3*:c.937C>T nonsense variant, we have 1 very strong evidence for pathogenicity (null variant in a gene where loss of function is a known mechanism of disease, PVS1), 1 moderate criterion (the mutant allele is absent from 461 control genomes, PM2), and 1 supporting evidence (demonstrated cosegregation in multiple affected members of a family, PP1). Collectively, these 3 lines of evidence allow us to classify *PAX3*:c.937C>T as pathogenic.

We found the mutant *DBE^RE^* allele in all DBE cats of the Dutch line (Rociri Elvis founder), but not in DBE cats of the Topaz lineage. This clearly indicates genetic heterogeneity of the feline DBE and warrants further studies to unravel additional causal variants for other forms of the DBE trait. It is not clear how far the *PAX3*-related genetic defect has already spread within the others feline DBE lineages. As *PAX3* is required for several key steps in neural development and based on data from mice, homozygosity for this allele will most likely result in embryonic or fetal lethality. Therefore, the mating of 2 heterozygous *PAX3*:c.937C>T cats is not recommended in order to avoid the accidental production of an embryo homozygous for this allele. Additionally, mating a carrier with a wild-type animal is also not recommended to prevent the birth of blue-eyed deaf cats.

Moreover, in adherence to the German Animal Protection Law, the breeding of animals with defective organ systems is explicitly prohibited, a criterion met by the *PAX3*-associated deafness described in this study. Considering that the Dutch and Topaz alleles have contributed to the establishment of multiple DBE catteries across several breeds, we strongly advocate implementation of the *PAX3* variant testing for all DBE cats. This will help the breeders in selection of suitable mating partners and production of healthy offspring.

In conclusion, we describe a *PAX3*-related auditory–pigmentary disorder in domestic cats. WGS revealed the heterozygous *PAX3*:c.937C>T variant as a potential and highly plausible underlying defect. However, further studies are required to evaluate the exact functional impact of this variant. Our data will allow genetic testing to avoid the unintentional breeding of further deaf kittens and provide a potential spontaneous animal model for the human WS.

## Supplementary Material

jkae131_Supplementary_Data

## Data Availability

WGS data can be accessed on the European Nucleotide Archive with the project ID PRJEB64577 and sample accessions SAMEA114193917 (WGS 1) and SAMEA114193918 (WGS 2). All genomes of the 99 Lives Cat Genome Consortium are deposited in the NCBI Short Read Archive. Supplemental material available at G3 online.
