# Correction to: *PAX3* haploinsufficiency in Maine Coon cats with dominant blue eyes and hearing loss resembling the human Waardenburg syndrome

**DOI:** 10.1093/g3journal/jkag156

**Published:** 2026-06-23

This is a correction to: Gabriela Rudd Garces, Daniela Farke, Martin J Schmidt, Anna Letko, Katja Schirl, Marie Abitbol, Tosso Leeb, Leslie A Lyons, Gesine Lühken, *PAX3* haploinsufficiency in Maine Coon cats with dominant blue eyes and hearing loss resembling the human Waardenburg syndrome, *G3 Genes|Genomes|Genetics*, Volume 14, Issue 9, September 2024, jkae131, https://doi.org/10.1093/g3journal/jkae131

The email address provided for co-corresponding author Gabriela Rudd Garces in the originally published version of this manuscript is no longer active.

This author can be contacted at gabriela.ruddgarces@gmx.de

These details have been corrected only in this correction notice to preserve the version of record.

