# Supplementary material for: PAX3 haploinsufficiency in Maine Coon cats with dominant blue eyes and hearing loss resembling the human Waardenburg syndrome
Source: G3 (Bethesda). 2024 Jun 13;14(9):jkae131. doi: 10.1093/g3journal/jkae131 (PMC11373664; doi:10.1093/g3journal/jkae131)

**Figure S1.** Pictures of two females green-eyed littermates showing white spotting phenotype. These cats are from the Dutch line of German origin, and are wildtype for the *PAX3*:c.937C>T variant. Both were heterozygous for the *w*^s^ allele causing white spotting. They correspond to animal IDs 3 and 16 in Table S4, respectively.


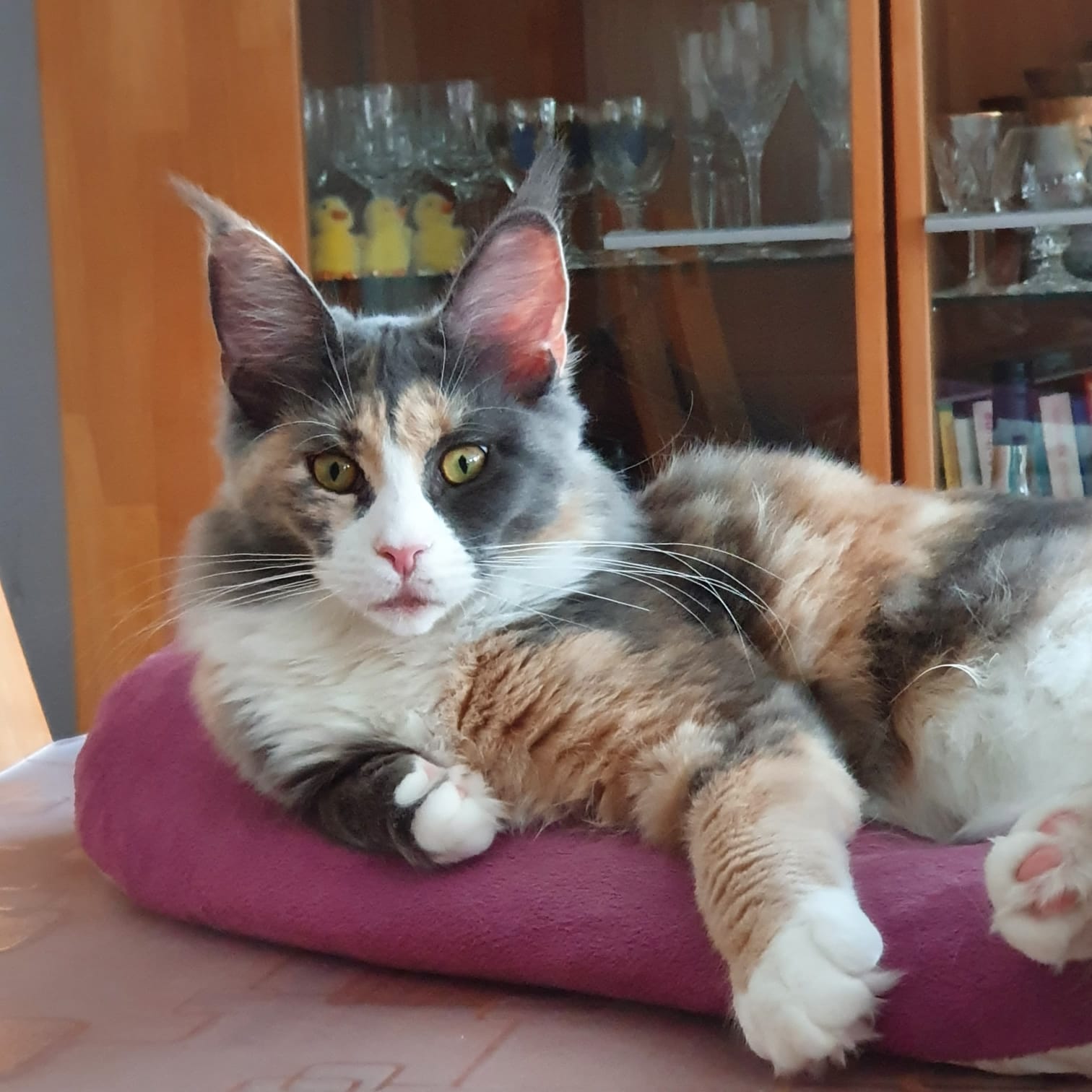


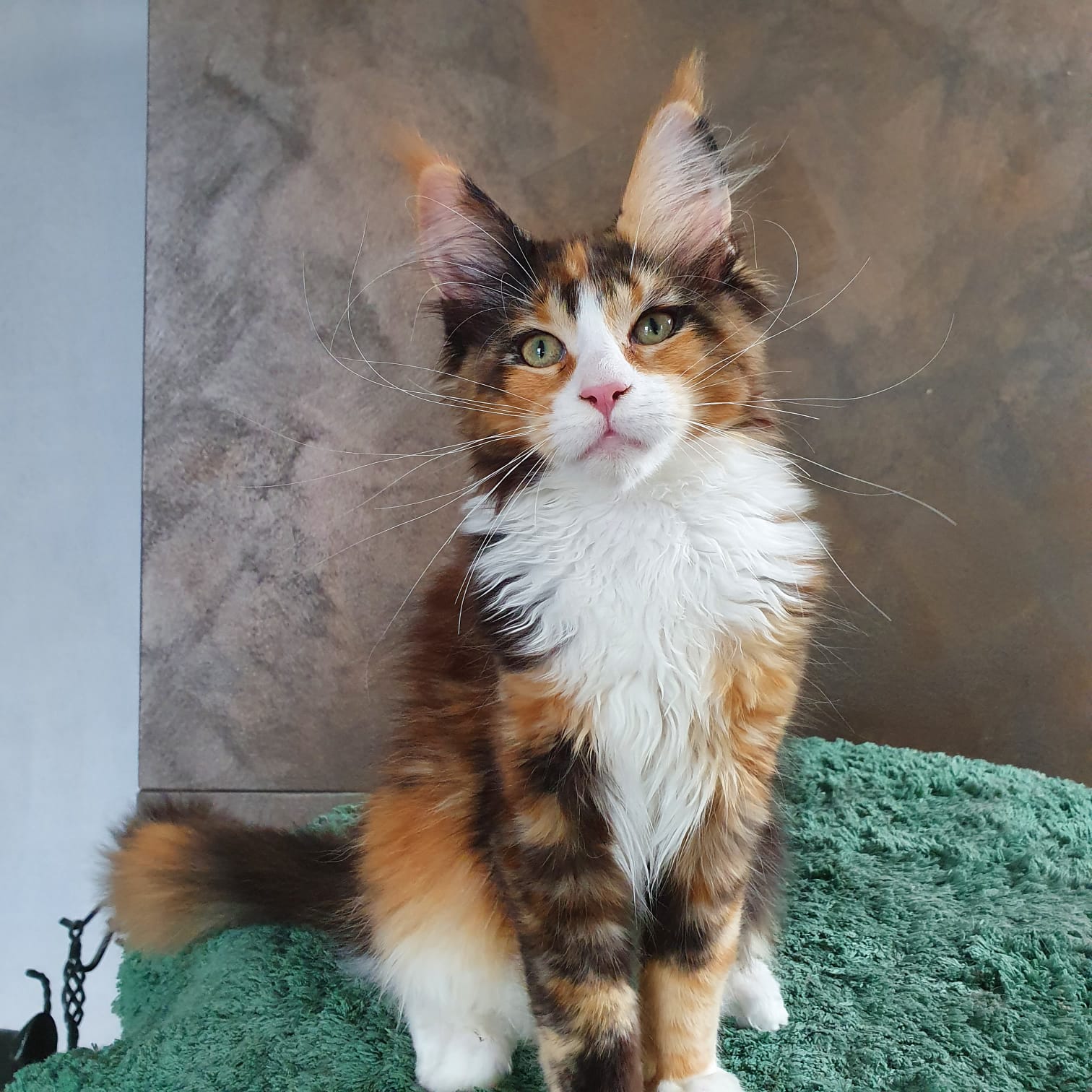


**Figure S2**. Picture of DBE male kitten without white spotting. This cat is from the Dutch line of Italian origin. It was heterozygous for the *PAX3*:c.937C>T variant and wildtype for the white spotting alleles. While this cat did not show any visible white spotting phenotype, several other heterozygous *PAX3*^+/-^ cats exhibited facial white spotting. It corresponds to animal ID 41 in Table S4.


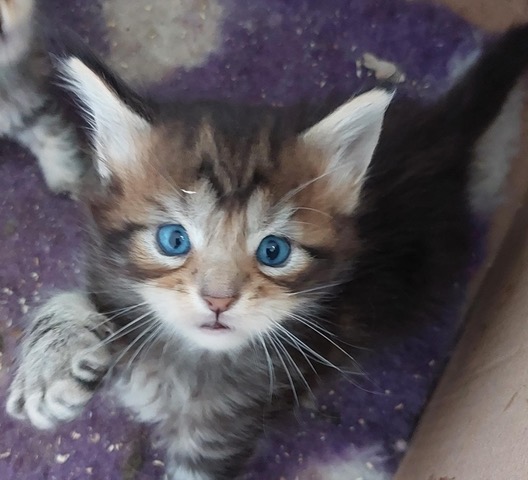

Supplement: jkae131_Supplementary_Data [file jkae131_supplementary_data.zip › File_S1_G3-2024-405131.docx]
